# Dynamics of fibrinogen in acute phases of trauma

**DOI:** 10.1186/s40560-016-0199-3

**Published:** 2017-01-20

**Authors:** Mineji Hayakawa

**Affiliations:** Emergency and Critical Care Center, Hokkaido University Hospital, N14W5 Kita-ku, Sapporo, 060-8648 Japan

**Keywords:** Coagulopathy, Disseminated intravascular coagulation, Fibrinolysis, Massive bleeding, Transfusion, Fibrinogen, Trauma

## Abstract

Fibrinogen is a unique precursor of fibrin and cannot be compensated for by other coagulation factors. If plasma fibrinogen concentrations are insufficient, hemostatic clots cannot be formed with the appropriate firmness. In severe trauma patients, plasma fibrinogen concentrations decrease earlier and more frequently than other coagulation factors, predicting massive bleeding and death. We review the mechanisms of plasma fibrinogen concentration decrease, which include coagulation activation-induced consumption, hyper-fibrino(geno)lysis-induced degradation, and dilution by infusion/transfusion. Understanding the mechanisms of plasma fibrinogen concentration decrease in severe trauma patients is crucial.

## Background

Fibrinogen is a glycopeptide that facilitates the formation of blood clots. It is synthesized in hepatocytes, with a molecular weight of 340 kDa [1, 2]. The plasma fibrinogen concentration is 1.5–4.0 g/L (as measured using the Clauss method), the highest level among other coagulation factors [1, 2]. As a unique precursor of fibrin, fibrinogen cannot be compensated for by other coagulation factors; if fibrinogen levels are insufficient in bleeding situations, fibrin clots for hemostasis cannot be formed with appropriate firmness [1, 2]. Furthermore, fibrinogen also acts as the ligand for glycoprotein IIb/IIIa receptors, found on the platelet surface, thereby accelerating platelet aggregation, similar to the role of the von Willebrand factor [2, 3]. In cases of thrombocytopenia, clot strength increases in direct proportion to plasma fibrinogen concentration, independent of platelet count [4]. Therefore, in acute phases of severe trauma, where bleeding control is important, fibrinogen plays a central role in hemostasis.

## Fibrinogen level in acute phases of trauma

In cases of severe trauma, depleted plasma fibrinogen levels are frequently observed before dilution by infusion [5–9]. Furthermore, plasma fibrinogen levels deteriorate more frequently and earlier than other routine coagulation parameters (prothrombin time, activated partial thromboplastin time, and platelet count) in severe trauma patients [5]. In a Japanese multicenter retrospective study, 25% of severe trauma patients (Injury Severity Score ≥ 16) had decreased plasma fibrinogen concentrations on arrival at the emergency department [6]. Critical (≤1.0 g/L) and abnormal (1.0–1.8 g/L) fibrinogen levels were also reported in 21 and 44% of severe trauma patients who required massive transfusions, respectively [8]. Decreased plasma fibrinogen levels on arrival at the emergency department are an independent predictor of massive transfusion requirement and death in severe trauma patients [5–9].

Although decreased plasma fibrinogen levels on arrival at the emergency department are an important risk factor of poor outcomes, the plasma fibrinogen concentration threshold considered as critically low has not been well-established in trauma patients. A decade ago, guidelines suggested that plasma fibrinogen concentrations of 1.0 g/L represented the critical threshold in bleeding patients [10]. However, recent guidelines have suggested that concentrations should be maintained over 1.5–2.0 g/L in severe trauma patients [11]. Furthermore, several retrospective studies indicated that fibrinogen levels ≤1.9 g/L on emergency department admission were independent predictors for massive bleeding and death [6, 12]. Based on these findings, the appropriate critical plasma fibrinogen threshold will be 2.0 g/L.

## Mechanisms of plasma fibrinogen decrease

**Fig. 1:**
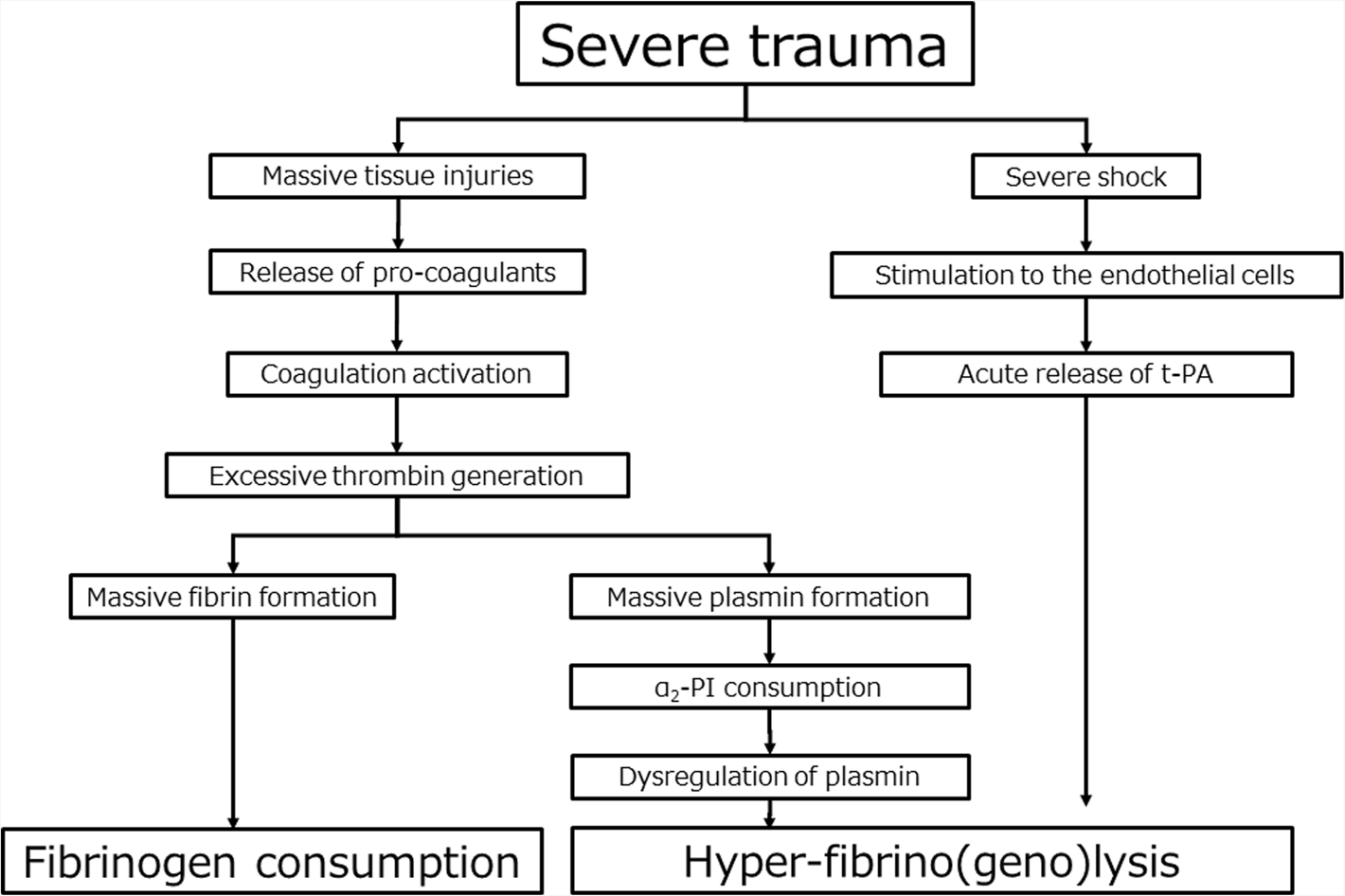
Mechanisms of plasma fibrinogen concentration decrease by severe trauma itself. t-PA, tissue-plasminogen activator; α_2_-PI, α_2_-plasmin inhibitor

Since, plasma fibrinogen concentrations decrease earlier and faster than other coagulation factors in severe trauma patients [5, 9, 13], elucidating the responsible mechanisms is of particular interest. There are three proposed mechanisms for plasma fibrinogen decrease: (1) coagulation activation-induced consumption, (2) hyper-fibrino(geno)lysis-induced degradation, and (3) dilution by infusion/transfusion. Both coagulation activation-induced consumption and hyper-fibrino(geno)lysis-induced degradation are caused by severe trauma itself (Fig. 1).

### Coagulation activation-induced consumption

**Fig. 2:**
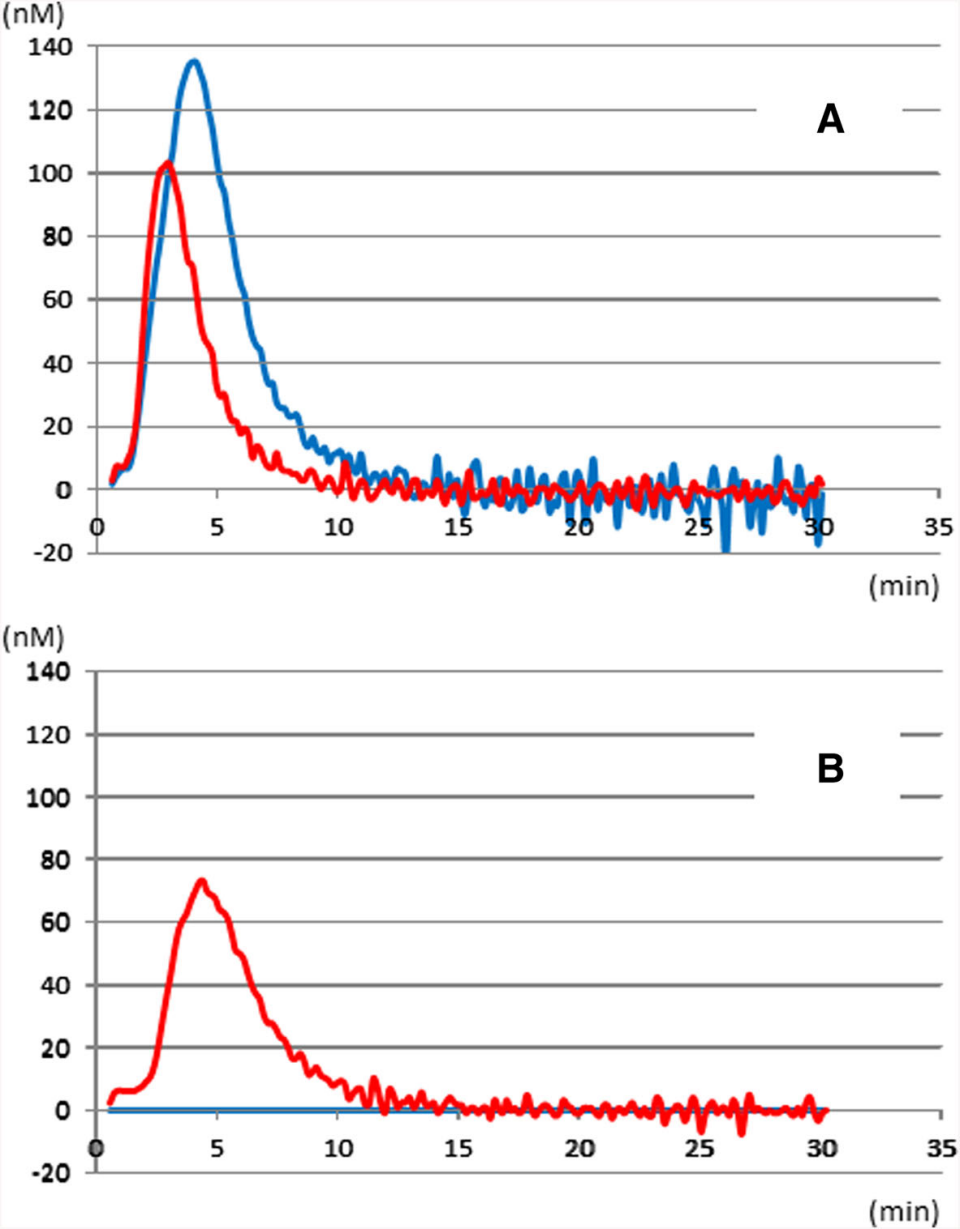
Spontaneous thrombin generation in severe trauma cases. **a** Stimulated thrombin generation curve in control (*blue*) and trauma (*red*) groups. Although thrombin generation is lower in the trauma group than the control group, time to thrombin generation initiation and time to peak thrombin generation are shorter in trauma patients compared to control, suggesting coagulation activation. **b** Non-stimulated thrombin generation curve in the trauma (*red*) group. Spontaneous thrombin generation was not observed in the control group, demonstrating the presence of circulating pro-coagulants in the trauma group. This figure was adapted from [33] with permission from Wolters Kluwer Health, Inc.

Following trauma, and particularly blunt trauma complicated by severe tissue injury, massively injured tissues accelerate spontaneous thrombin generation, induced by pro-coagulants in plasma (Fig. 2) [14–17]. These circulating pro-coagulants are known as damage-associated molecular patterns (DAMPs) [18–26] and microparticles [27–32] released from injured organs/tissues.

Extracellular DNA and DNA-binding proteins are the principal DAMPs that comprise the pro-coagulants detected in severe trauma patients. Histone and histone-complexed DNA fragments have been detected in the systemic circulation just after trauma [18, 19]. Furthermore, early release of high mobility group box nuclear protein 1 (HMGB-1), which is a non-histonal DNA binding protein [20–23], and mitochondrial DNA [24–26] are also observed just after trauma. Elevation of the levels of these DAMPs is related with inflammation, coagulation activation, massive bleeding, and poor outcome [18–26].

Various cell-derived microparticles have been detected during the acute phase of severe trauma [27–32]. Platelet-derived microparticles are well-known pro-coagulants in the acute phase of trauma [27–29]. Furthermore, leukocyte-, erythrocyte-, and endothelial cell-derived microparticles are also released into the systemic circulation in the acute phase of trauma (29, 30). In animal models of brain trauma, brain-derived microparticles that expressed neuronal or glial cell markers were detected in the systemic circulation [31, 32]. These microparticles were confirmed to express not only pro-coagulant phosphatidylserine but also tissue factor on their membranes [29, 31, 32].

These DAMPs and microparticles are released into the plasma from injured organs/tissues just after trauma and activate the coagulation cascade following the conversion of fibrinogen to fibrin. Furthermore, massive DAMPs and microparticles induce consumptive coagulopathy [23, 30, 31].

### Hyper-fibrino(geno)lysis-induced degradation

The newly formed fibrin is subsequently converted to a D-dimer and fibrin/fibrinogen degradation products (FDP) via degradation by hyper-fibrino(geno)lysis, which is a combination of fibrinolysis and fibrinogenolysis [12, 16].

Coagulopathy caused by severe trauma is known as disseminated intravascular coagulation with fibrinolytic phenotype and is characterized by hyper-fibrino(geno)lysis, which is a combination of fibrinolysis and fibrinogenolysis [5, 12, 14–17, 33–42]. Hyper-fibrino(geno)lysis is caused by the acute release of tissue-plasminogen activator (t-PA) and by coagulation activation.

#### Shock-induced fibrino(geno)lysis

Weibel-Palade bodies are storage granules found in systemic vascular endothelial cells and normally contain t-PA [43–45]. The t-PA found in Weibel-Palade bodies are released into circulation during tissue hypoperfusion (severe shock), in a process known as *acute release of t-PA* [43, 44]. This rapid t-PA release from endothelial cells activates the conversion of plasminogen to plasmin and induces hyper-fibrino(geno)lysis [12, 16, 46, 47]. Shock-induced hyper-fibrino(geno)lysis are confirmed as lysis of clot formed in its test tube by thromboelastometry, such as ROTEM®, and is a predictor for massive bleeding and death [48–53]. Typical hyper-fibrino(geno)lysis detected via thromboelastometry is infrequent and is associated with very high mortality rates [48, 51, 53].

#### Coagulation activation-induced fibrino(geno)lysis

In severe trauma, elevations in D-dimer and FDP levels are frequently observed and are complicated with coagulopathy, regardless of severe shock [6, 12, 16, 54–59]. Although severe head trauma is not generally complicated with shock, trauma-induced coagulopathy is frequently observed with this type of injury [54, 56–58]. Kushimoto et al. [54] have indicated that patients with severe head trauma and poor outcomes have elevated fibrinogen degradation product (a kind of FDP) levels and markedly decreased fibrinogen levels on emergency department admission. Elevated fibrinogen degradation product levels correlated with elevated plasmin-α_2_ plasmin inhibitor complex levels are reported to result in hyper-fibrino(geno)lysis [54]. Many other studies reported the presence of D-dimer and FDP in not only cases of isolated head trauma [54, 56–59] but also torso trauma regardless of shock [6, 12, 16]. Furthermore, another investigation reported that hyper-fibrino(geno)lysis in severe head trauma is not directly related to shock [60]. This type of hyper-fibrino(geno)lysis is not caused by the shock-related acute release of t-PA, but by massive tissue injuries-induced coagulation activation [54, 60]. Some reports have indicated that high levels of circulating pro-coagulants are related to high levels of D-dimer and t-PA [19, 23]. In an animal study, tissue factor administration induced coagulation activation and reactive hyper-fibrino(geno)lysis without shock [55]. In severe trauma, especially blunt trauma, massively injured tissues accelerate thrombin generation [14–17]. This excessive thrombin generation not only induces fibrin formation, but also simultaneously promotes plasmin generation and the consumption of α_2_-plasmin inhibitor [36, 41, 61]. Low levels of the α_2_-plasmin inhibitor trigger the release of plasmin and induce hyper-fibrino(geno)lysis.

### Dilution by infusion/transfusion therapy

Severe trauma-related depletion of plasma fibrinogen levels is observed before and upon emergency department admission, and levels continue to decrease after blood infusion/transfusion therapy initiation [5–9]. We showed that plasma fibrinogen levels deteriorate earlier and more frequently than other routine coagulation parameters (prothrombin time, activated partial thromboplastin time, and platelet count) in severe trauma patients after the initiation of infusion/transfusion therapies [5]. Furthermore, even in massive bleeding cases without severe tissue injuries and shock, plasma fibrinogen is more easily decreased to critical levels than other coagulation factors by infusion/transfusion therapy in the absence of plasma administration [13, 62]. Therefore, fibrinogen and/or plasma should be aggressively supplemented in patients with severe trauma [63–65].

### Evaluation and treatment for fibrinogen consumption and hyper-fibrino(geno)lysis in clinical settings

In clinical settings, we usually evaluate the plasma fibrinogen level by conducting measurements in a laboratory. Although knowledge of plasma fibrinogen levels is required for prompt treatment of patients with severe trauma, the laboratory measurements of fibrinogen levels usually takes more than 30 min. Therefore, the early evaluation of fibrinogen levels is considered important [4, 7, 48, 50, 51, 53, 66]. Thromboelastometry has been widely used for early evaluation of fibrinogen level in severe trauma patients [4, 7, 48, 50, 51, 53]. However, the technique requires 10 to 15 min to measure fibrinogen levels, thus, limiting its application [4, 7, 48, 50, 51, 53]. Another technique used for early evaluation of fibrinogen levels is by measuring the levels using a compact whole blood coagulation analyzer (CG02N; A&T Corporation, Kanagawa, Japan) [66, 67]. The analyzer can rapidly measure fibrinogen concentrations in whole blood within 2 min, allowing for a rapid and accurate diagnosis of fibrinogen deficiency [66, 67]. In any case, it is important to promptly evaluate fibrinogen deficiency and to supplement fibrinogen and/or plasma in severe trauma patients [63–65].

Early evaluation of hyper-fibrino(geno)lysis is difficult in clinical settings. Shock-induced hyper-fibrino(geno)lysis is diagnosed via thromboelastometry [48–53]. However, the technique requires more than 30 min to evaluate hyper-fibrino(geno)lysis [48–53]. Furthermore, coagulation activation-induced fibrino(geno)lysis cannot be evaluated based on thromboelastometry [68]. However, note that elevated D-dimer levels are reflected not only in shock-induced hyper-fibrino(geno)lysis but also in coagulation activation-induced fibrino(geno)lysis [6, 68]. Therefore, hyper-fibrino(geno)lysis may be evaluated via evaluation of D-dimer levels in patients with acute phase trauma [6, 68]. When hyper-fibrino(geno)lysis is observed or speculated in acute phase of trauma, anti-fibrinolytic drug (tranexamic acid) should be administrated as soon as possible [69].

## Conclusions

Although fibrinogen is an important factor in hemostasis, it is easily decreased to critical levels in severe trauma patients [5–9, 13, 62]. To avoid hyper-fibrino(geno)lysis, which deteriorates fibrinogen concentrations, early administration of an anti-fibrinolytic drug (e.g., tranexamic acid) improves severe trauma patients’ mortality rates [69]. Aggressive supplementation of fresh frozen plasma is effective in countering decreased fibrinogen concentrations [63]. Studies evaluating effective fibrinogen supplementation in severe trauma are currently underway [70, 71].

## Abbreviations

DAMPs: Damage-associated molecular patterns
FDP: Fibrin/fibrinogen degradation products
t-PA: Tissue-plasminogen activator

## References

[1] Sorensen B, Larsen OH, Rea CJ, Tang M, Foley JH, Fenger-Eriksen C (2012). Fibrinogen as a hemostatic agent. Semin Thromb Hemost.

[2] Lowe GD, Rumley A, Mackie IJ (2004). Plasma fibrinogen. Ann Clin Biochem.

[3] Levy JH, Welsby I, Goodnough LT (2014). Fibrinogen as a therapeutic target for bleeding: a review of critical levels and replacement therapy. Transfusion.

[4] Lang T, Johanning K, Metzler H, Piepenbrock S, Solomon C, Rahe-Meyer N (2009). The effects of fibrinogen levels on thromboelastometric variables in the presence of thrombocytopenia. Anesth Analg.

[5] Hayakawa M, Gando S, Ono Y, Wada T, Yanagida Y, Sawamura A (2015). Fibrinogen level deteriorates before other routine coagulation parameters and massive transfusion in the early phase of severe trauma: a retrospective observational study. Semin Thromb Hemost.

[6] Hayakawa M, Maekawa K, Kushimoto S, Kato H, Sasaki J, Ogura H (2016). High D-dimer levels predict a poor outcome in patients with severe trauma, even with high fibrinogen levels on arrival: a multicenter retrospective study. Shock.

[7] Schochl H, Cotton B, Inaba K, Nienaber U, Fischer H, Voelckel W (2011). FIBTEM provides early prediction of massive transfusion in trauma. Crit Care.

[8] Inaba K, Karamanos E, Lustenberger T, Schochl H, Shulman I, Nelson J (2013). Impact of fibrinogen levels on outcomes after acute injury in patients requiring a massive transfusion. J Am Coll Surg.

[9] Floccard B, Rugeri L, Faure A, Saint Denis M, Boyle EM, Peguet O (2012). Early coagulopathy in trauma patients: an on-scene and hospital admission study. Injury.

[10] O’Shaughnessy DF, Atterbury C, Bolton Maggs P, Murphy M, Thomas D, Yates S (2004). Guidelines for the use of fresh-frozen plasma, cryoprecipitate and cryosupernatant. Br J Haematol.

[11] Spahn DR, Bouillon B, Cerny V, Coats TJ, Duranteau J, Fernandez-Mondejar E (2013). Management of bleeding and coagulopathy following major trauma: an updated European guideline. Crit Care.

[12] Sawamura A, Hayakawa M, Gando S, Kubota N, Sugano M, Wada T (2009). Disseminated intravascular coagulation with a fibrinolytic phenotype at an early phase of trauma predicts mortality. Thromb Res.

[13] Hiippala S (1998). Replacement of massive blood loss. Vox Sang.

[14] Gando S, Nakanishi Y, Kameue T, Nanzaki S (1995). Soluble thrombomodulin increases in patients with disseminated intravascular coagulation and in those with multiple organ dysfunction syndrome after trauma: role of neutrophil elastase. J Trauma.

[15] Gando S, Kameue T, Matsuda N, Hayakawa M, Ishitani T, Morimoto Y (2002). Combined activation of coagulation and inflammation has an important role in multiple organ dysfunction and poor outcome after severe trauma. Thromb Haemost.

[16] Hayakawa M, Sawamura A, Gando S, Kubota N, Uegaki S, Shimojima H (2011). Disseminated intravascular coagulation at an early phase of trauma is associated with consumption coagulopathy and excessive fibrinolysis both by plasmin and neutrophil elastase. Surgery.

[17] Engelman DT, Gabram SG, Allen L, Ens GE, Jacobs LM (1996). Hypercoagulability following multiple trauma. World J Surg.

[18] Abrams ST, Zhang N, Manson J, Liu T, Dart C, Baluwa F (2013). Circulating histones are mediators of trauma-associated lung injury. Am J Respir Crit Care Med.

[19] Johansson PI, Windelov NA, Rasmussen LS, Sorensen AM, Ostrowski SR (2013). Blood levels of histone-complexed DNA fragments are associated with coagulopathy, inflammation and endothelial damage early after trauma. J Emerg Trauma Shock.

[20] Giannoudis PV, Mallina R, Harwood P, Perry S, Sante ED, Pape HC (2010). Pattern of release and relationship between HMGB-1 and IL-6 following blunt trauma. Injury.

[21] Wang XW, Karki A, Du DY, Zhao XJ, Xiang XY, Lu ZQ (2015). Plasma levels of high mobility group box 1 increase in patients with posttraumatic stress disorder after severe blunt chest trauma: a prospective cohort study. J Surg Res.

[22] Wang XW, Karki A, Zhao XJ, Xiang XY, Lu ZQ (2014). High plasma levels of high mobility group box 1 is associated with the risk of sepsis in severe blunt chest trauma patients: a prospective cohort study. J Cardiothorac Surg.

[23] Cohen MJ, Brohi K, Calfee CS, Rahn P, Chesebro BB, Christiaans SC (2009). Early release of high mobility group box nuclear protein 1 after severe trauma in humans: role of injury severity and tissue hypoperfusion. Crit Care.

[24] Zhang Q, Raoof M, Chen Y, Sumi Y, Sursal T, Junger W (2010). Circulating mitochondrial DAMPs cause inflammatory responses to injury. Nature.

[25] Yamanouchi S, Kudo D, Yamada M, Miyagawa N, Furukawa H, Kushimoto S (2013). Plasma mitochondrial DNA levels in patients with trauma and severe sepsis: time course and the association with clinical status. J Crit Care.

[26] Simmons JD, Lee YL, Mulekar S, Kuck JL, Brevard SB, Gonzalez RP (2013). Elevated levels of plasma mitochondrial DNA DAMPs are linked to clinical outcome in severely injured human subjects. Ann Surg.

[27] Park MS, Xue A, Spears GM, Halling TM, Ferrara MJ, Kuntz MM (2015). Thrombin generation and procoagulant microparticle profiles after acute trauma: A prospective cohort study. J Trauma Acute Care Surg.

[28] Park MS, Owen BA, Ballinger BA, Sarr MG, Schiller HJ, Zietlow SP (2012). Quantification of hypercoagulable state after blunt trauma: microparticle and thrombin generation are increased relative to injury severity, while standard markers are not. Surgery.

[29] Nekludov M, Mobarrez F, Gryth D, Bellander BM, Wallen H (2014). Formation of microparticles in the injured brain of patients with severe isolated traumatic brain injury. J Neurotrauma.

[30] Matijevic N, Wang YW, Wade CE, Holcomb JB, Cotton BA, Schreiber MA (2014). Cellular microparticle and thrombogram phenotypes in the Prospective Observational Multicenter Major Trauma Transfusion (PROMMTT) study: correlation with coagulopathy. Thromb Res.

[31] Tian Y, Salsbery B, Wang M, Yuan H, Yang J, Zhao Z (2015). Brain-derived microparticles induce systemic coagulation in a murine model of traumatic brain injury. Blood.

[32] Yasui H, Donahue DL, Walsh M, Castellino FJ, Ploplis VA (2016). Early coagulation events induce acute lung injury in a rat model of blunt traumatic brain injury. Am J Physiol Lung Cell Mol Physiol.

[33] Hayakawa M, Gando S, Ono Y, Wada T, Yanagida Y, Sawamura A (2015). Noble-Collip drum trauma induces disseminated intravascular coagulation but not acute coagulopathy of trauma-shock. Shock.

[34] Yanagida Y, Gando S, Sawamura A, Hayakawa M, Uegaki S, Kubota N (2013). Normal prothrombinase activity, increased systemic thrombin activity, and lower antithrombin levels in patients with disseminated intravascular coagulation at an early phase of trauma: comparison with acute coagulopathy of trauma-shock. Surgery.

[35] Gando S, Kameue T, Nanzaki S, Hayakawa T, Nakanishi Y (1997). Participation of tissue factor and thrombin in posttraumatic systemic inflammatory syndrome. Crit Care Med.

[36] Gando S, Nanzaki S, Kemmotsu O (1999). Coagulofibrinolytic changes after isolated head injury are not different from those in trauma patients without head injury. J Trauma.

[37] Oshiro A, Yanagida Y, Gando S, Henzan N, Takahashi I, Makise H (2014). Hemostasis during the early stages of trauma: comparison with disseminated intravascular coagulation. Crit Care.

[38] Brohi K, Cohen MJ, Ganter MT, Matthay MA, Mackersie RC, Pittet JF (2007). Acute traumatic coagulopathy: initiated by hypoperfusion: modulated through the protein C pathway?. Ann Surg.

[39] Brohi K, Cohen MJ, Ganter MT, Schultz MJ, Levi M, Mackersie RC (2008). Acute coagulopathy of trauma: hypoperfusion induces systemic anticoagulation and hyperfibrinolysis. J Trauma.

[40] Cohen MJ, Call M, Nelson M, Calfee CS, Esmon CT, Brohi K (2012). Critical role of activated protein C in early coagulopathy and later organ failure, infection and death in trauma patients. Ann Surg.

[41] Gando S, Tedo I, Kubota M (1992). Posttrauma coagulation and fibrinolysis. Crit Care Med.

[42] Gando S, Nanzaki S, Sasaki S, Kemmotsu O (1998). Significant correlations between tissue factor and thrombin markers in trauma and septic patients with disseminated intravascular coagulation. Thromb Haemost.

[43] Lowenstein CJ, Morrell CN, Yamakuchi M (2005). Regulation of Weibel-Palade body exocytosis. Trends Cardiovasc Med.

[44] Kooistra T, Schrauwen Y, Arts J, Emeis JJ (1994). Regulation of endothelial cell t-PA synthesis and release. Int J Hematol.

[45] Huber D, Cramer EM, Kaufmann JE, Meda P, Masse JM, Kruithof EK (2002). Tissue-type plasminogen activator (t-PA) is stored in Weibel-Palade bodies in human endothelial cells both in vitro and in vivo. Blood.

[46] Gando S, Otomo Y (2015). Local hemostasis, immunothrombosis, and systemic disseminated intravascular coagulation in trauma and traumatic shock. Crit Care.

[47] Gando S, Hayakawa M (2016). Pathophysiology of trauma-induced coagulopathy and management of critical bleeding requiring massive transfusion. Semin Thromb Hemost.

[48] Levrat A, Gros A, Rugeri L, Inaba K, Floccard B, Negrier C (2008). Evaluation of rotation thrombelastography for the diagnosis of hyperfibrinolysis in trauma patients. Br J Anaesth.

[49] Kutcher ME, Cripps MW, McCreery RC, Crane IM, Greenberg MD, Cachola LM (2012). Criteria for empiric treatment of hyperfibrinolysis after trauma. J Trauma Acute Care Surg.

[50] Theusinger OM, Baulig W, Seifert B, Muller SM, Mariotti S, Spahn DR (2015). Changes in coagulation in standard laboratory tests and ROTEM in trauma patients between on-scene and arrival in the emergency department. Anesth Analg.

[51] Theusinger OM, Wanner GA, Emmert MY, Billeter A, Eismon J, Seifert B (2011). Hyperfibrinolysis diagnosed by rotational thromboelastometry (ROTEM) is associated with higher mortality in patients with severe trauma. Anesth Analg.

[52] Kashuk JL, Moore EE, Sawyer M, Wohlauer M, Pezold M, Barnett C (2010). Primary fibrinolysis is integral in the pathogenesis of the acute coagulopathy of trauma. Ann Surg.

[53] Schochl H, Frietsch T, Pavelka M, Jambor C (2009). Hyperfibrinolysis after major trauma: differential diagnosis of lysis patterns and prognostic value of thrombelastometry. J Trauma.

[54] Kushimoto S, Shibata Y, Yamamoto Y (2003). Implications of fibrinogenolysis in patients with closed head injury. J Neurotrauma.

[55] Hayakawa M, Gando S, Ieko M, Honma Y, Homma T, Yanagida Y (2013). Massive amounts of tissue factor induce fibrinogenolysis without tissue hypoperfusion in rats. Shock.

[56] Goodnight SH, Kenoyer G, Rapaport SI, Patch MJ, Lee JA, Kurze T (1974). Defibrination after brain-tissue destruction: a serious complication of head injury. N Engl J Med.

[57] Saggar V, Mittal RS, Vyas MC (2009). Hemostatic abnormalities in patients with closed head injuries and their role in predicting early mortality. J Neurotrauma.

[58] Tian HL, Chen H, Wu BS, Cao HL, Xu T, Hu J (2010). D-dimer as a predictor of progressive hemorrhagic injury in patients with traumatic brain injury: analysis of 194 cases. Neurosurg Rev.

[59] Hijazi N, Abu Fanne R, Abramovitch R, Yarovoi S, Higazi M, Abdeen S (2015). Endogenous plasminogen activators mediate progressive intracerebral hemorrhage after traumatic brain injury in mice. Blood.

[60] Lustenberger T, Talving P, Kobayashi L, Barmparas G, Inaba K, Lam L (2010). Early coagulopathy after isolated severe traumatic brain injury: relationship with hypoperfusion challenged. J Trauma.

[61] Risberg B, Medegard A, Heideman M, Gyzander E, Bundsen P, Oden M (1986). Early activation of humoral proteolytic systems in patients with multiple trauma. Crit Care Med.

[62] McLoughlin TM, Fontana JL, Alving B, Mongan PD, Bunger R (1996). Profound normovolemic hemodilution: hemostatic effects in patients and in a porcine model. Anesth Analg.

[63] Holcomb JB, Tilley BC, Baraniuk S, Fox EE, Wade CE, Podbielski JM (2015). Transfusion of plasma, platelets, and red blood cells in a 1:1:1 vs a 1:1:2 ratio and mortality in patients with severe trauma: the PROPPR randomized clinical trial. JAMA.

[64] Schochl H, Schlimp CJ (2014). Trauma bleeding management: the concept of goal-directed primary care. Anesth Analg.

[65] Schlimp CJ, Schochl H (2014). The role of fibrinogen in trauma-induced coagulopathy. Hamostaseologie.

[66] Hayakawa M, Gando S, Ono Y, Mizugaki A, Katabami K, Maekawa K (2015). Rapid evaluation of fibrinogen levels using the CG02N whole blood coagulation analyzer. Semin Thromb Hemost.

[67] Ogawa S, Tanaka KA, Nakajima Y, Nakayama Y, Takeshita J, Arai M (2015). Fibrinogen measurements in plasma and whole blood: a performance evaluation study of the dry-hematology system. Anesth Analg.

[68] Raza I, Davenport R, Rourke C, Platton S, Manson J, Spoors C (2013). The incidence and magnitude of fibrinolytic activation in trauma patients. J Thromb Haemost.

[69] Shakur H, Roberts I, Bautista R, Caballero J, Coats T, collaborators C-t (2010). Effects of tranexamic acid on death, vascular occlusive events, and blood transfusion in trauma patients with significant haemorrhage (CRASH-2): a randomised, placebo-controlled trial. Lancet.

[70] Steinmetz J, Sorensen AM, Henriksen HH, Lange T, Larsen CF, Johansson PI (2016). Pilot randomized trial of fibrinogen in trauma haemorrhage (PRooF-iTH): study protocol for a randomized controlled trial. Trials.

[71] Maegele M, Zinser M, Schlimp C, Schochl H, Fries D (2015). Injectable hemostatic adjuncts in trauma: fibrinogen and the FIinTIC study. J Trauma Acute Care Surg.

